# Supplementary material for: Serotypes, Antimicrobial Resistance Profiles, and Virulence Factors of Salmonella Isolates in Chinese Edible Frogs (Hoplobatrachus rugulosus) Collected from Wet Markets in Hong Kong
Source: Foods. 2023 Jun 1;12(11):2245. doi: 10.3390/foods12112245 (PMC10252521; doi:10.3390/foods12112245)
Supplement: Supplementary file 1 [file foods-12-02245-s001.zip › Supplementary Table S1.pdf]

Serotypes, Antimicrobial Resistance Profiles, and Virulence Factors of *Salmonella* Isolates in Chinese Edible Frogs (*Hoplobatrachus rugulosus*) Collected from Wet Markets in Hong Kong

Sara Boss et al., Foods, 2023.

Table S1. Antibiotic efflux pump genes and target alteration resistance mechanisms detected by RGI software in 67 *Salmonella* from Chinese edible frogs (*Hoplobatrachus rugulosus*) collected from wet markets in Hong Kong.

Resistance mechanism	Antimicrobial class(es)	ARG	Isolates (n)	Isolates (%)	Remarks
efflux	aminocoumarin, aminoglycosides	*baeR*	67	100.0	
efflux	aminocoumarin, aminoglycosides	*cpxA*	67	100.0	
efflux	disinfectants and antiseptics	*qacE*D1	13	19.4	in isolates F31, F33, F35, F36, F40, F48, F49, F50, F51, F52, F93, F43, F82
efflux	fluoroquinolones	*emrA*	67	100.0	
efflux	fluoroquinolones	*emrB*	67	100.0	
efflux	fluoroquinolones	*emrD*	67	100.0	
efflux	fluoroquinolones	*qepA2*	1	1.5	in isolate F93
efflux	fluoroquinolones, cephalosporins, penams, cephamycins	*acrA*	67	100.0	
efflux	fluoroquinolones, cephalosporins, penams, cephamycins	*acrB*	67	100.0	
efflux	fluoroquinolones, cephalosporins, penams, cephamycins	*acrD*	67	100.0	
efflux	fluoroquinolones, cephalosporins, penams, cephamycins	*acrB*	67	100.0	
efflux	fluoroquinolones, cephalosporins, penams, cephamycins	*acrD*	67	100.0	

Resistance mechanism	Antimicrobial class(es)	ARG	Isolates (n)	Isolates (%)	Remarks
efflux	fluoroquinolones, diaminopyrimidines, phenicols	*rsmA*	67	100.0	
efflux	macrolides, fluoroquinolones, penams	*crp*	67	100.0	
efflux	multidrug	*kdpD*	67	100.0	
efflux	multidrug	*kdpE*	67	100.0	
efflux	multidrug	*kdpF*	67	100.0	
efflux	multidrug	*mdtB*	67	100.0	
efflux	multidrug	*mdtC*	67	100.0	
efflux	multidrug	*mdtK*	67	100.0	
efflux	multidrug	*mdtM*	67	100.0	
efflux	multidrug	*golS*	67	100.0	
efflux	multidrug	*mdsA*	67	100.0	
efflux	multidrug	*mdsB*	67	100.0	
efflux	multidrug	*mdsC*	67	100.0	
efflux	multidrug	*soxR*	67	100.0	
efflux	multidrug	*soxS*	67	100.0	
efflux	multidrug	*marA*	67	100.0	
efflux	multidrug	*marR*	67	100.0	
efflux	nitroimidazole	*msbA*	67	100.0	
efflux	phenicols	*cmlA5*	1	1.5	in isolate F93
efflux	tetracycline, disinfectants and antiseptics	*mdfA*	67	100.0	
efflux	nucleosides, disinfectants and antiseptics	*leuO*	58	87.0	
target alteration	fosfomycin	*glpT*	67	100.0	
target alteration	fosfomycin	*uhpT*	67	100.0	
target alteration	peptide antibiotics	*bacA*	67	100.0	
target alteration	peptide antibiotics	*pmrF*	67	100.0	
target alteration	quinolones	*gyrA* (S83F)	1	1.5	in isolate F77
