# Supplementary material for: Serotypes, Antimicrobial Resistance Profiles, and Virulence Factors of Salmonella Isolates in Chinese Edible Frogs (Hoplobatrachus rugulosus) Collected from Wet Markets in Hong Kong
Source: Foods. 2023 Jun 1;12(11):2245. doi: 10.3390/foods12112245 (PMC10252521; doi:10.3390/foods12112245)
Supplement: Supplementary file 1 [file foods-12-02245-s001.zip › Supplementary Table S2.pdf]

Serotypes, Antimicrobial Resistance Profiles, and Virulence Factors of *Salmonella* Isolates in Chinese Edible Frogs (*Hoplobatrachus rugulosus*) Collected from Wet Markets in Hong Kong

Sara Boss et al., Foods, 2023.

Table S2. title; Antimicrobial susceptibility determined by disk diffusion of 67 *Salmonella* isolated from Chinese edible frogs (*Hoplobatrachus rugulosus*) from wet markets in Hong Kong.

ID	Serotype	ST	AM	AMC	CZ	CTX	FEP	NA	CIP	GM	K	STR	SXT	FOS	AZM	F/M	TE	C
F7	*S.* Bareilly	909	R	S	S	S	S	S	I	S	S	R	S	S	R	S	R	S
F9	*S.* Bareilly	909	R	S	S	S	S	S	I	S	S	I	S	S	R	S	R	S
F61	*S.* Bareilly	909	R	S	S	S	S	S	I	S	S	I	S	S	R	S	R	S
F62	*S.* Bareilly	909	S	S	S	S	S	S	I	S	S	S	S	S	S	S	S	S
F64	*S.* Bareilly	909	R	S	S	S	S	S	I	S	S	R	S	S	R	S	R	S
F1	*S.* Braenderup	311	S	S	S	S	S	I	I	S	S	S	S	S	S	S	S	S
F2	*S.* Braenderup	311	S	S	S	S	S	S	I	S	S	R	S	S	S	S	S	S
F3	*S.* Braenderup	311	S	S	S	S	S	R	I	S	S	S	S	S	S	S	S	S
F78	*S.* Hvittingfoss	446	S	S	S	S	S	I	R	S	S	S	S	S	S	S	R	S
F83	*S.* Hvittingfoss	446	S	S	S	S	S	I	I	S	S	S	S	S	S	S	R	S
F85	*S.* Hvittingfoss	446	S	S	S	S	S	I	I	S	S	S	S	S	S	S	R	S
F29	*S.* Newport	31	R	S	S	S	S	S	I	S	S	S	S	S	S	S	R	S
F32	*S.* Newport	31	R	S	S	S	S	S	I	S	S	S	S	S	S	S	R	S
F38	*S.* Newport	31	S	S	S	S	S	S	I	S	S	S	S	S	S	S	S	S
F39	*S.* Newport	31	R	S	S	S	S	S	I	S	S	S	S	S	S	S	R	S
F44	*S.* Newport	31	R	S	S	S	S	S	I	S	S	S	S	S	S	S	R	S
F55	*S.* Newport	31	S	S	S	S	S	S	I	S	S	S	R	S	S	S	R	S
F56	*S.* Newport	31	S	S	S	S	S	S	I	S	S	S	R	S	S	S	R	S

ID	Serotype	ST	AM	AMC	CZ	CTX	FEP	NA	CIP	GM	K	STR	SXT	FOS	AZM	F/M	TE	C
F67	*S.* Newport	31	S	S	S	S	S	I	R	S	S	S	R	S	S	S	R	S
F40	*S.* Newport	45	R	R	R	S	S	I	R	S	R	R	S	S	S	S	R	R
F43	*S.* Newport	45	R	R	R	S	S	I	I	S	R	R	S	S	S	S	R	R
F48	*S.* Newport	45	R	I	S	S	S	I	R	S	R	S	S	S	S	S	R	R
F49	*S.* Newport	45	R	R	R	S	S	I	I	S	R	R	S	S	S	S	R	R
F50	*S.* Newport	45	R	R	R	S	S	I	I	S	R	R	S	S	S	S	R	R
F51	*S.* Newport	45	R	R	R	I	S	I	R	R	R	R	S	S	S	S	R	R
F52	*S.* Newport	45	R	R	R	S	I	I	R	S	R	R	S	S	S	S	R	R
F77	*S.* Newport	46	S	S	S	S	S	R	I	S	S	S	S	S	S	S	R	S
F11	*S.* Saintpaul	50	S	S	S	S	S	R	R	S	S	S	S	S	S	S	R	S
F13	*S.* Saintpaul	50	S	S	S	S	S	R	I	S	S	S	S	S	S	S	S	S
F15	*S.* Saintpaul	50	S	S	S	S	S	R	I	S	S	S	S	S	S	S	R	S
F21	*S.* Saintpaul	50	S	S	S	S	S	R	I	S	S	S	S	S	S	S	S	S
F23	*S.* Saintpaul	50	S	S	S	S	S	R	I	S	S	S	S	S	S	S	S	S
F24	*S.* Saintpaul	50	S	S	S	S	S	R	I	S	S	S	S	S	S	S	S	S
F25	*S.* Saintpaul	50	S	S	S	S	S	R	I	S	S	S	S	S	S	S	S	S
F26	*S.* Saintpaul	50	S	S	S	S	S	R	I	S	S	S	S	S	S	S	S	S
F27	*S.* Saintpaul	50	S	S	S	S	S	R	I	S	S	S	S	S	S	S	S	S
F28	*S.* Saintpaul	50	S	S	S	S	S	R	R	S	S	S	S	S	S	S	R	S
F30	*S.* Saintpaul	50	S	S	S	S	S	R	I	S	S	S	S	S	S	S	S	S
F65	*S.* Saintpaul	50	S	S	S	S	S	R	R	S	S	S	S	S	S	S	R	I
F66	*S.* Saintpaul	50	S	S	S	S	S	R	I	S	S	S	S	S	S	S	S	S
F73	*S.* Saintpaul	50	S	S	S	S	S	R	R	S	S	S	S	S	S	S	R	S
F75	*S.* Saintpaul	50	S	S	S	S	S	R	R	S	S	S	S	S	S	S	R	S
F79	*S.* Saintpaul	50	S	S	S	S	S	R	R	S	S	S	S	S	S	S	R	S
F80	*S.* Saintpaul	50	S	S	S	S	S	R	I	S	S	S	S	S	S	S	S	S
F92	*S.* Saintpaul	50	S	S	S	S	S	R	R	S	S	S	S	S	S	S	R	S
F59	*S.* Saintpaul	50	S	S	S	S	S	R	I	S	S	S	S	S	S	S	R	S
F81	*S.* Saintpaul	50	S	S	S	S	S	R	I	S	S	S	S	S	S	S	S	S
F58	*S.* Saintpaul	50	S	S	S	S	S	R	I	S	S	S	S	S	S	S	S	S
F93	*S.* Saintpaul	50	R	S	S	S	S	R	R	S	S	S	S	S	R	S	R	S
F4	*S.* Stanley	29	S	S	S	S	S	S	I	S	S	S	S	S	S	I	S	S
F5	*S.* Stanley	29	S	S	S	S	S	S	I	S	S	S	S	S	S	S	S	S
F6	*S.* Stanley	29	S	S	S	S	S	S	I	S	S	S	S	S	S	S	S	S
F8	*S.* Stanley	29	S	S	S	S	S	S	I	S	S	S	S	S	S	S	S	S
F60	*S.* Stanley	29	S	S	S	S	S	S	I	S	S	S	S	S	S	S	S	S
F63	*S.* Stanley	29	S	S	S	S	S	S	I	S	S	S	S	S	S	S	S	S
F57	*S.* Stanley	29	S	S	S	S	S	S	I	S	S	S	S	S	S	S	S	S
F12	*S.* Wandsworth	1498	S	S	S	S	S	I	I	S	S	S	S	S	S	S	S	S
F68	*S.* Wandsworth	1498	S	S	S	S	S	I	I	S	S	S	S	S	S	S	S	S
F70	*S.* Wandsworth	1498	S	S	S	S	S	I	I	S	S	S	S	S	S	S	S	I

ID	Serotype	ST	AM	AMC	CZ	CTX	FEP	NA	CIP	GM	K	STR	SXT	FOS	AZM	F/M	TE	C
F82	*S.* Wandsworth	1498	S	S	S	S	S	I	R	S	S	S	R	S	S	S	R	S
F86	*S.* Wandsworth	1498	S	S	S	S	S	I	I	S	S	S	S	S	S	S	S	S
F31	*S.* Wandsworth	1498	S	S	S	S	S	S	I	S	S	S	R	S	S	S	R	S
F33	*S.* Wandsworth	1498	S	S	S	S	S	S	I	S	S	S	R	S	S	S	R	S
F35	*S.* Wandsworth	1498	S	S	S	S	S	S	I	S	S	S	R	S	S	S	R	S
F36	*S.* Wandsworth	1498	S	S	S	S	S	S	I	S	S	S	R	S	S	S	R	S
F14	*S.* Wandsworth	1498	S	S	S	S	S	S	I	S	S	S	S	S	S	S	R	S
F45	*S.* Wandsworth	1498	S	S	S	S	S	S	I	S	S	S	S	S	S	S	S	S

AM, ampicillin; AMC, amoxicillin/clavulanic acid; AZM, azithromycin; C, chloramphenicol; CZ, cefazolin; CIP, ciprofloxacin; CTX, cefotaxime; FEP, cefepime; F/M, nitrofurantoin; FOS, fosfomycin; GM, gentamicin; I, intermediate; K, kanamycin; NA, nalidixic acid; R, resistant; S, susceptible; ST, sequence type; STR, streptomycin; SXT, sulfamethoxazole-trimethoprim; TE, tetracycline.
